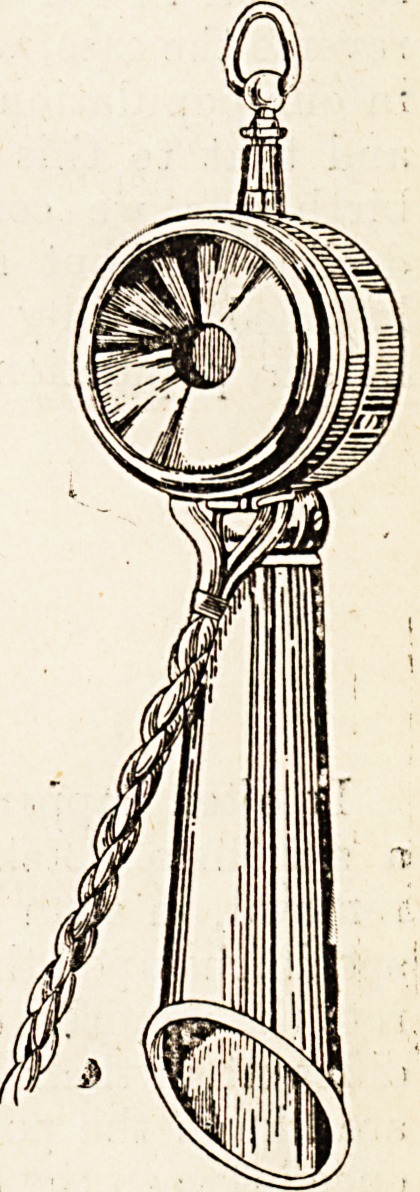# The Hygiene of the Public Telephone

**Published:** 1907-10-05

**Authors:** 


### Public Health and Hygiene.

### THE HYGIENE OF THE PUBLIC TELEPHONE.

There can be little doubt that there is a real danger of the spread of such diseases as diphtheria, phthisis, and syphilis, by the use of that form of telephone which is at present general in London and in other places in England. The speaker has to hold his mouth very close to, if not in actual contact with, the speaking tube—much closer than was necessary with the older instrument in which one handle held both earpiece and mouthpiece. Quite apart from the physical necessity of sitting or standing in one particular place opposite the instrument, instead of being able to sit down comfortably in a chair and hold the speaking apparatus in any position one chose, as was formerly the case, the inferiority of the present machine from a sanitary point of view is obvious. It is true that bacteriological examinations have shown that in many there is no dangerous contamination by bacteria, but the fact that some instruments are not contaminated does not prove that there is no danger from others; in business offices, in public buildings, in public telephone call offices, and in similar places where numbers of different persons of all classes come and go, using the same machine, the present form of instrument is a source of danger to health, and it is the duty of medical men to have it remedied.

It is, however, of little use to insist on a defect unless, at the same time, a remedy can be given. This remedy is at hand in the case of the telephone. It is well known that, with a properly sensitive receiver, there is no necessity at all to speak directly into it. Many of the old-pattern instruments allowed of the mouthpiece being held above or below the mouth, or even turned backwards behind the head, and yet what the speaker said was clearly audible to the listener at the other end of the wire. The difficulty of that particular pattern of machine was that it could only be sensitised by means of a battery, which used to be enclosed within the telephone case; every telephone thus had to have its own sensitising battery—a source of great expense. The present machines are sensitised from the current in the main telephone wire—a great advantage to the telephone company. A Mr. Holmström, of Stockholm, has overcome the difficulty now, however, and has invented an inexpensive form of instrument in which the sensitising current is from the main wire, and yet the speaking-tube and ear-piece are in one. As will be seen from the above illustration, it is not merely unnecessary, but actually impossible to get the speaking-tube anywhere near the mouth at the same time as the ear-piece is on the ear. This makes no difference to the audibility of what one says; the speaking-tube may be held pointing upwards, forwards, downwards, or backwards, it is all the same, and it conveys quite clearly what one says, whatever direction one speaks in. The instrument is a very great advantage over the present one from the point of view of convenience of attitude in carrying on a conversation; it is a still greater advantage from the hygienic point of view—it cannot become contaminated by the speaker, and, even if contaminated in some way, it will not infect a subsequent speaker. It is to be hoped that Mr. Holmström's telephone will be tried at once by the National Telephone Company, the Post Office Telephone authorities, and others; and if it is found as serviceable as it seems to be, it, or some modification of it, should be introduced as soon as possible, in order to remove what is at present a source of public danger.

## Figures and Tables

**Figure f1:**